# Chronic pain among children with cerebral palsy attending a Ugandan tertiary hospital: a cross-sectional study

**DOI:** 10.1186/s12887-021-02928-1

**Published:** 2021-10-18

**Authors:** Emma Nsalazi Bambi, Angelina Kakooza Mwesige, Hervé Monka Lekuya, Philip Kasirye, Richard Idro

**Affiliations:** 1 Department of Paediatrics and Child Health, Makerere University, Kampala, Uganda; 2 Department of Surgery, Makerere University, Kampala, Uganda

**Keywords:** Chronic pain, Cerebral palsy, Co- morbid conditions, Motor function, Associated factors

## Abstract

**Background:**

Children with cerebral palsy (CP) frequently experience chronic pain. The burden and severity of such pain is often underestimated in relation to their other impairments. Recognition and awareness of this chronic pain among children with CP constitute the cornerstone for caretakers and clinicians to improve the quality of life of those children. This study aimed to determine the prevalence of chronic pain among children with CP, and the factors associated.

**Methods:**

A cross-sectional study of children with CP, aged 2–12 years, attending the CP rehabilitation clinic and Pediatric Neurology Clinic at Mulago Hospital, Uganda from November 2017 to May 2018. A detailed history and clinical examination were performed and the co-morbidities were determined. CP was classified using the Gross Motor Function Classification System (GMFCS), Manual Ability Classification System, Communication Function Classification System (CFCS), and the Eating and Drinking Ability Classification System (EDACS) and documented with the level of impairment in the different domains. Pain was assessed by using the revised Face, Legs, Activity, Consolability, Cry pain scale.

**Results:**

A total of 224 children with CP were enrolled. The prevalence of chronic pain was 64.3%. The majority had spastic bilateral CP (77.8%), moderate pain lasting over 6 months, and none of them was on long-term pain management. Epilepsy (60.9%), behavioral problem (63.2%), hearing impairment (66,7%), learning problem (67,6%), dental caries (75%), gastro-esophageal reflux (75%), sleep disorders (79.5%), vision impairment (80%), and malnutrition (90%) were co- morbid conditions of chronic pain in children with CP in this study. The factors independently associated with chronic pain among children with CP were the GMFCS level IV & V, CFCS level IV & V, EDACS level IV & V, female children, and caretaker aged more than 30 years.

**Conclusions:**

Two-thirds of children with CP attending rehabilitation in this hospital had chronic pain. None was receiving pain management. Chronic pain was associated with the presence of multiple co-morbidities and more severe disability. Rehabilitation and care programs for children with CP should include assessment of pain in routine care and provide interventions for pain relief in children with CP even at an early age.

## Background

Cerebral palsy in children and adolescents is a major social disability, and affecting their quality of life, and largely of their caretakers. There is a growing recognition that pain is a significant problem in children and adolescents with cerebral palsy (CP) [1]. About 65 to78% of children with CP are reported by their caretakers to experience pain [2]. Research has shown that children with CP who have pain participate less in everyday situations, experience lower quality of life than those without pain, and interferes with sleep [2]. Moreover, children with CP who experience pain are more likely to struggle with anxiety, frustration,fear,depression, and behavior disorders such as aggression [3].Self-report is considered the gold standard for pain assessment due to pain’s subjective and individual in nature; this is particularly challenging for children with CP because some have a speech impairment, which makes it difficult to communicate their experience and others have such severe cognitive impairment that proxy reports are necessary [4]. Research supports this alternate approach to data collection or clinical assessment, as Engel and colleagues found the agreement between parent and child report of pain occurrence was 93% [2]. Currently, there is very little literatures about how common are chronic pain among children with CP in sub-Saharan African, especially in Uganda, where there is a high prevalence of CP among children resulting from several peri-natal causes. There is little knowledge about the factors that predispose to this pain and their associated complications. Recognition and awareness of chronic pain and the burden of associated factors of this pain among children with CP could help us to provide an accurate assessment, and plan for better interventions to improve the quality of life of those children. The study aimed to determine the prevalence of chronic pain in children with CP, to describe the severity and Co- morbidities of pain, and determine the factors associated with such pain.

## Methods

### Study design and setting

This cross-sectional study was carried out in the Cerebral Palsy Rehabilitation Clinic and the Pediatric Neurology Clinic at Mulago National Referral Hospital, Kampala, Uganda, between November 2017and May 2018.

### Study participants

Cerebral palsy is a neuro-developmental condition comprising a group of permanent disorders of movement and posture that are attributed to non-progressive injury that occurred in the developing fetal or infant brain [5]. Study participants were Children of 2–12 years old with a documented diagnosis of CP attending regularly the above-mentioned clinics for their follow-up, accompanied by a caretaker. Caretaker was considered as the mother, father, grandparent or guardian of a child with cerebral palsy who spends most of time with the child in a day and has been living with her/him for a period of at least 3 months. The latter was responsible for providing most of the material and emotional requirements to the child for at least 3 months. Only patients whose parents or caretakers provide informed consent were included. Participants who came with caretakers who were unable to provide adequate information about the child on the study or declined to provide informed consent were excluded in the study.

### Study procedure

The study team consisted of medical personnel working within the above-mentioned Paediatrics clinics with additionally trained research assistants to do the enrolment, consent, history, and physical examination. The detailed clinical history as well as co- morbidities was obtained from the parents or caretakers; the child was examined to obtain the features and complications of CP. The patient’s level of physical functioning was assessed using the Gross Motor Function Classification system (GMFCS). The GMFCS has levels I-V with level I indicating a good level of functioning while level V is the worst depicting severe disabled. The ability of hand functioning was also assessed using the manual ability classification system (MACS) which has also levels I to V. This was followed by assessing the child’s communication ability using the communication function classification system (CFCS). The eating and drinking ability classification system (EADACS) was assessed as well, knowing that EADACS has levels I-V.

### Measurement of pain in children with CP

Pain was assessed using the revised face, legs, activity, cry and consolability (rFLACC) scale for children aged between 2 and 12 years old. The rFLACC scale questionnaires for children with CP were filled to determine the pain and the severity of pain. This instrument has been recognized to be reproducible, valid, and reliable in assessing pain in children with CP not able to self-report pain. The internal consistency is excellent with a Cronbach’s alpha of 0.9023 and 0.9753(two raters).A test–retest showed excellent intra- rater reliability with an intra-class correlation (ICC) of 0.97530 [6]. The r-FLACC is a useful tool for assessing pain in children with Cognitive impairment due to its ease to use in a clinical setting and its use of both core and individual pain behaviors [6]. It has been used in Africa as well, precisely in study done by Shabana et al. in Egypt for assessing pain in children with CP [7].

### Data analysis

#### Descriptive analysis

Descriptive statistics were used to explore baseline characteristics of the participants and caretakers; they were presented as means and Medians, ranges and IQR. Results were presented in tables, graphs and text.

#### Bivariate and multivariate analysis

A logistic regression model was used to find out factors that significantly determine having chronic pain among children with CP. Variables whose *p* value of the Un adjusted Odd Ratio (bivariable level) was less than 0.2 were considered at multivariable logistic regression analysis to find out factors that significantly determine having chronic pain among children with CP. Significance was set at *P* Value of ≤0.05.

### Ethical approval

Approval was sought from the Department of Paediatrics and Child Health of Makerere University College of Health Sciences, as well as the School of Medicine Research and Ethics Committee (SOMREC).

## Results

Between November 2017 and May 2018, a total of 234 children with CP attended the Cerebral Palsy Rehabilitation Clinic or General Paediatric Neurology Clinics in Mulago Hospital were invited to participate in the study; 4 caretakers decline to consent, 2 were outside the required age range of 2-12 years, 4 were unable to participate due to other reasons and 224 were recruited. The majority of participants were male (62.5%), with an overall median age of 3.6 years (Table 1).Most of the caretakers were literate married Christian mothers,with no formal employment, living in a family size of more than 4 people (Table 2).

**Table 1:** Socio-demographic and medical history of children with CP

Characteristics	Frequency (***N*** = 224)	Percentage (%)
**Age (in years)**		
Median (IQR)	3.6 (2.5–5.6)	–
**Sex**		
Male	140	62.5
Female	84	37.5
**Pregnancy/Delivery**		
Normal	123	54.9
Difficulty	84	37.5
Not known	17	7.6
**Delivery**		
Term	190	84.8
Preterm	9	4.0
Not known	25	11.2
**Birth weight (in kilograms)**		
Mean(SD)	3.3 (3.1,3.4)	–
**Neonatal problems**		
Yes	178	79.5
No	46	20.5

**Table 2:** Socio-demographic characteristics of the main caretakers of children with CP

Characteristics	Frequency	Percentage (%)
**Age (in years)**		
Median (IQR)	30 (27,38)	
**Sex**		
Male	22	9.8
Female	202	90.2
**Relationship to child**		
Mother	179	79.9
Father	18	8.0
Others	27	12.1
**Employment status**		
Employed	85	37.9
Unemployed or others	139	62.1
**Education status**		
No schooling	18	8.0
Primary	80	35.7
Secondary	87	38.9
Tertiary	9	17.4
**Marital status**		
Married	163	72.7
Single	27	12.1
Other^a^	34	15.2
**Religion**		
Christian	183	81.7
Muslim	41	18.3
**Family size**		
< 4 members	91	40.6
≥ 4 members	133	59.4

^a^includes separated, divorced and widowed

One hundred forty-four (144) of the 224 children presented with chronic pain giving a prevalence of 64.3% (Table 3). The highest percentage of children with chronic pain was observed with children with spastic bilateral CP (77.8%). None of those children with CP in chronic pain was on long-term pain management. Participants in level IV & V of GMFCS, MACS, CFCS, and EADCS had a high prevalence of chronic pain compare to participants in level I, II & III (Table 3). The majority of participants had moderate pain, but the duration of pain was more than 6 months (Table 4). Epilepsy,sleep disorders,impaired vision impaired hearing, dental caries, malnutrition, gastro esophageal reflux, behavioral problem, and Hearing problem were co- morbid conditions in this study (Table 5). Female children had at least two times the odds of having chronic pain compared to male children (OR, 95% CI:2.50,1.13–5.73,*p* = 0.024). Also, GMFCS was significantly associated with having chronic pain in this population of children with CP. Children who were in level IV & V of GMFCS had almost 4 times the odds of having chronic pain compared to those that were in either level I or II or III;{OR,95%CI:3.75(1.38–10.20), *p* = 0.009}. Also children who were in level IV or V of EADACS and CFCS had a higher risk of having chronic pain compared to those in level I or II or III{OR,95%CI:2.46(1.03–5.90), *p* = 0.044and3.32,1.37–8.04; *p* = 0.008 respectively}. The results also further revealed that children with older caretakers (≥ 30 years) were less likely to have chronic pain compared to those taken care of by caretakers less than 30 years{OR,95%CI:0.45(0.21–0.95),*p* = 0.03}(Table 6).

**Table 3:** Chronic pain according to clinical characteristics of children with CP

Characteristics	Frequency (N = 224)	Percentage (%)
**Chronic Pain**		
Yes	144	64.3
No	80	35.7
**Chronic pain according to the type of CP**		
Spastic bilateral	91/117	77.8
Spastic unilateral	12/24	50.0
Dyskinetic CP	27/55	49.1
Ataxic CP	4/7	57.1
Non classifiable	10/21	47.6
**Chronic pain according to GMFCS**		
Level I	1/14	7.1
Level II	2/14	14.3
Level III	5/13	38.5
Level IV	39/65	60.0
Level V	97/118	82.2
**Chronic pain according to MACS**		
Level I	4/27	14.8
Level II	7/27	25.9
Level III	10/19	52.6
Level IV	29/39	74.4
Level V	94/112	83.9
**Chronic pain according to CFCS**		
Level I	0/19	0
Level II	10/30	33.3
Level III	14/29	48.3
Level IV	26/35	74.3
Level V	94/111	84.7
**Chronic pain according to EADACS**		
Level I	8/31	25.8
Level II	9/35	25.9
Level III	30/47	63.8
Level IV	81/95	85.3
Level V	16/16	100

**Table 4:** Severity and duration of pain among children with CP

Severity of pain	Duration of pain- n (%)	
	3–6 months (***n*** = 33)	More than 6 months (***n*** = 111)
Moderate pain	28 (84.8)	64 (57.7)
Severe pain	5 (15.2)	47 (42.3)

**Table 5:** Co-morbid conditions among children with CP

Comorbidity	Chronic pain/N	Prevalence (%)	95%CI
**Illnesses**			
Epilepsy	84/138	60.9	48.6–75.4
Dental caries	12/16	75.0	38.8–100^
Malnutrition	10/11	90.9	43.6–100^
Gastro esophageal reflux disease	6/8	75.0	27.5–100^
**Physical functioning problems**			
Hearing Impairment	4/6	66.7	18.2–100^
Vision Impairment	16/20	80.0	45.7–100^
**Behavioral problem**			
Sleeping disorders	31/39	79.5	54.0–100^
Presence of aggressive behavior			
Yes	55/87	63.2	47.6–82.3
No	89/137	64.9	52.2–79.9
Plays with other children			
Yes	48//103	46.6	34.4–61.8
No	96/121	79.3	64.3–96.9
**Learning problem**			
Schooling	2/14	14.3	1.7–51.6
Not at School	142/210	67.6	57.0–79.7

**Table 6:** Factors independently associated with chronic pain among children with CP

Characteristics	Overall N = 224	Unadjusted OR (95% CI)	***p***-value	Adjusted OR (95%CI)	p-value
**Child’s gender**					
Male	140(62.5)	1.00	1.00		
Female	84(37.5)	1.67(0.93–2.98)	0.085	2.54(1.13–5.73)	**0.024**
**CP classification**					
Spastic bilateral	117(52.2)	1.00		1.00	
Spastic unilateral	24(10.7)	0.28(0.12–0.71)	0.007	0.80(0.25–2.53)	0.701
Dyskinetic CP	55(24.6)	0.27(0.14–0.55)	0.001	0.64(0.27–1.56)	0.328
Ataxic CP	7(3.1)	0.38(0.08–1.81)	0.225	1.45(0.15–13.71)	0.747
Non-classifiable	21(9.4)	0.26(0.10–0.68)	0.006	0.75(0.18–3.02)	0.681
**Classification**					
GMFCS					
Level I, II, & III	41(18.3)	1.00		1.00	
Level IV & V	183(81.7)	11.94(5.14–27.7)	< 0.001	3.75(1.38–10.20)	**0.009**
MACS					
Level I, II, & III	73(32.6)	1.00		1.00	
Level IV & V	151(67.4)	10.9(5.7–20.9)	< 0.001	2.54(0.99–6.5)	0.052
CFCS					
Level I, II, & III	78(34.8)	1.00		1.00	
Level IV & V	146(65.2)	10.4(5.5–19.7)	< 0.001	2.46(1.03–5.90)	**0.044**
EADACS					
Level I, II, & III	113(50.5)	1.00		1.00	
Level IV& V	111(49.5)	9.73(4.96–19.09)	< 0.001	3.32(1.37–8.04)	**0.008**
**Comorbidities**					
No	60(26.8)	1.00			
Yes	164(73.2)	0.96(0.52–1.78)	0.893		
**Main caregiver’s age**					
< 30	104(46.4)	1.00		1.00	
≥ 30	120(53.6)	0.62(0.35–1.07)	0.087	0.45(0.21–0.95)	**0.035**

## Discussion

This study set out to determine the prevalence of chronic pain and associated factors among children with CP. We found that 64.3% of these patients who had chronic pain with higher GMFCS levels involvement or severe disability were more at risk.

### Prevalence of chronic pain in children with cerebral palsy

The prevalence of chronic pain in this study is consistent with previous studies done in other countries such us in Malaysia in 2015 [8] and turkey in 2017 [9], where the prevalence of chronic pain in children with CP in both 2 studies was found to be 65%. Also, the prevalence of pain in children with CP in this study is similar to a previous report that indicated that around 60% of children with CP experience recurrent pain on a daily or weekly basis that significantly interferes with their daily Activities [10].

### Prevalence of chronic pain according to type of CP, GMFCS, MACS, CFCS, EADACS

In this study the most common type of CP with the highest prevalence of chronic pain was spastic bilateral CP. Knowing that children with spastic bilateral CP have more severe functional motor limitation and experiencing stiffness and contractures which usually predispose them to ongoing pain. A similar pattern was found in a study done by Badia et al. in 2014 where most participants with pain had bilateral spastic CP [1]. Participants with GMFCS,MACS,CFCS,EADACS level IV,V had the highest prevalence of pain in this study;these findings are concurring with a previous cross-sectional multi-centre European study done by Gibson et al. in 2009 that reported a higher frequency and severity of pain commonly found if the child is more severely impaired in terms of walking ability,hand function,feeding and communication difficulties, intellect and severe type of CP [2]. Another study done in Sweden in 2017 by Lena et al. indicated that children with CP with Level V in both Gross Motor Function Classification System (GMFCS) and Manual Ability Classification System (MACS) were associated with the highest prevalence of pain and level I with lowest prevalence of pain [11]. Furthermore, 2 other studies done by Parkinson et al. in 2013 [12] and Barney et al. in 2013 [13] discovered that pain frequency, intensity, and duration were significantly predicted by GMFCS level, being greater among children with a higher GMFCS level IV, V. Similarly, a study done in Turkey in 2017 [9], showed that children with GMFCS IV, V had a high percentage of getting pain compared to patients at GMFCS levels I, II, and III.

### Severity of pain

In this study majority of participants had moderate pain. A similar pattern was reported in a study done in Malaysia by Subhashini et al. in 2015 where patients with moderate pain were more than those with severe pain [5]. These findings are also similar to a previous study done in Spain that showed that persons with CP experience recurrent pain of moderate-intensity on a daily or weekly basis that significantly interferes with daily activities [10].

### Co-morbidities

Co-morbid problems in this study are similar to a previous study done by Badia et al. in2014, where epilepsy, hearing disability, visual disability, behavior problem, communication problem, feeding problem were the most common co-morbid conditions in children with pain and CP [1]. Another study done by Raymond et al. presented similar findings to ours where the commonest co-morbid conditions that parents reported in their children with pain and CP were a developmental delay, vision problem,learning problem, behavior problem, speech problem, seizure disorder,hearing impairment, and sleep disturbance [14].

### Factors associated with chronic pain in children with cerebral palsy

Chronic pain is a significant problem among children and adolescents and is associated with significant functional disability and other poor outcomes, including problems with school attendance and performance, disruptions in family life, appetite, sleep, and participation in enjoyable activities [15]. In this study, we found that gross motor function classification level IV &V, Communication function classification level IV&V, eating and drinking ability classification system level IV &V, female participants,and caretaker age of more than 30 years were independently significant factors associated with chronic pain. Indeed in the multi-centre European cross-sectional study done by Parkinson et al. in 2009, it was reported that a higher frequency and severity of pain was common if the child was more severely impaired in terms of walking ability, hand function, feeding and communication difficulties, intellect and CP type [2]. Knowing that patients with GMFCS, EADACS, and CFCS level IV &V usually have a severe disability in term of motor impairment and persons with severe impairment tend to have more spasticity, which causes ongoing pain. Also in this study, we found that female participants had at least two times the odds of having chronic pain compared to the male child. A similar pattern was reported by Christine et al, in 2004 where the female was reported to have a greater frequency of pain compare to the male [16]. Children with older caretakers (≥ 30 years) were less likely to have chronic pain. This may be explained by the fact that older caretakers have much more experience of recognition of pain, and may be giving more support to relieve this chronic pain. In a study done by Abigail M et al. in 2021, it showed that caretakers were able to report different types of pain but also used non pharmacologic approaches such as changing positions, massage as well as medication to relieve pain in children with CP [17].

### Study limitations

This study was a relatively small sample size. The nature of the study design could only describe chronic pain among children with CP and not give details about different causes of pain. The type and location of pain could not be clearly ascertained when dealing with children with speech and cognitive impairments. Although we used validated tools from previous studies, children with CP in sub-Saharan African countries could have benefited from an adjusted tool regarding local realities in terms of diagnosis, management and follow up of chronic pain in children with CP in our settings.

## Conclusions

Approximately two-thirds of children with CP attending rehabilitation in this tertiary hospital experience chronic pain. The majority of these children have moderate pain without long-term pain management. Co-morbid conditions such as epilepsy sleep disorders, impairment of vision, and impairment of hearing, dental caries, malnutrition, gastro esophageal reflux, behavioral problem, and learning problem are the common co-morbid conditions among children with CP presenting with chronic pain. In addition severe disability in terms of GMFCS, CFCS, EADACS (level IV&V), and female gender were significantly and independently associated with chronic pain among children with CP. However having had an older caretakers (≥ 30 years) was associated with better pain control. We recommend a routine pain assessment, and its management to improve the quality of life of children with CP, especially when they have co-morbid conditions.

## Data Availability

The datasets used and/or analyzed during the current study are not publicly available due to legal and ethical reasons but are available from the corresponding author on reasonable request.

## Abbreviations

CP: Cerebral palsy
CFCS: Communication function classification system
EADACS: Eating and drinking ability classification system
GMFCS: Gross- motor function classification system
MACS: Manual ability classification system
rFLACC: Revised face, legs, activity, consolability, cries pain scale
